# THE OLDER POPULATION LIVING WITH AND PROVIDING CARE TO GRANDCHILDREN, BY NATIVITY: 2013-2017

**DOI:** 10.1093/geroni/igz038.992

**Published:** 2019-11-08

**Authors:** Bashiruddin Ahmed, Andrew Roberts, Matthew Spence, Wan He, Laquitta Walker

**Affiliations:** 1 U.S. Census Bureau Population Division, Washington, District of Columbia, United States; 2 United States Census Bureau, Washington, District of Columbia, United States; 3 U.S. Census Bureau, Washington, District of Columbia, United States

## Abstract

The rapid growth of the older population in the United States combined with changing living arrangements, marital status, and employment, increases the importance of multi-generational ties for the well-being of families. The U.S. Census Bureau’s earlier reports on grandparents living with grandchildren mostly focused on the background characteristics of all grandparents without classifying them by nativity. This study expands on the research by presenting data for both native- and foreign-born grandparents aged 60 and older who live with and provide care to their grandchildren under 18. Data for this study come from the 2013-2017 American Community Survey 5-year estimates. Among native-born grandparents living with grandchildren, the majority were females, aged 60-69, White alone, non-Hispanic or Latino, married, high school graduate or higher, had no disability, lived in a household that was owned, uninsured, not in labor force, and not in poverty. The foreign-born grandparents were similar in most characteristics except for race component and educational attainment. Key findings include: • Among total older population, 14.3 percent of the foreign-born lived with grandchildren, compared with 4.1 percent of the native-born. • Among co-resident grandparents, the native-born (36 percent) were more likely to be caregivers, compared with the foreign-born (14 percent). • The proportions of co-resident grandparents widely vary by race and Hispanic origin. • Among grandparent caregivers, over 50 percent had been responsible for grandchildren for 5 years or more, while 14 percent for less than a year. • Both native- and foreign-born grandparents show declining patterns of care-giving by age.

